# The Genetic Correlation between Height and IQ: Shared Genes or Assortative Mating?

**DOI:** 10.1371/journal.pgen.1003451

**Published:** 2013-04-04

**Authors:** Matthew C. Keller, Christine E. Garver-Apgar, Margaret J. Wright, Nicholas G. Martin, Robin P. Corley, Michael C. Stallings, John K. Hewitt, Brendan P. Zietsch

**Affiliations:** 1 Department of Psychology and Neuroscience, University of Colorado Boulder, Boulder, Colorado, United States of America; 2 Institute for Behavioral Genetics, University of Colorado Boulder, Boulder, Colorado, United States of America; 3 Department of Psychiatry, University of Colorado School of Medicine, Denver, Colorado, United States of America; 4 Genetic Epidemiology Laboratory, Queensland Institute for Medical Research, Brisbane, Queensland, Australia; 5 School of Psychology, University of Queensland, Brisbane, Queensland, Australia; University of Chicago Howard Hughes Medical Institute, United States of America

## Abstract

Traits that are attractive to the opposite sex are often positively correlated when scaled such that scores increase with attractiveness, and this correlation typically has a genetic component. Such traits can be genetically correlated due to genes that affect both traits (“pleiotropy”) and/or because assortative mating causes statistical correlations to develop between selected alleles across the traits (“gametic phase disequilibrium”). In this study, we modeled the covariation between monozygotic and dizygotic twins, their siblings, and their parents (total N = 7,905) to elucidate the nature of the correlation between two potentially sexually selected traits in humans: height and IQ. Unlike previous designs used to investigate the nature of the height–IQ correlation, the present design accounts for the effects of assortative mating and provides much less biased estimates of additive genetic, non-additive genetic, and shared environmental influences. Both traits were highly heritable, although there was greater evidence for non-additive genetic effects in males. After accounting for assortative mating, the correlation between height and IQ was found to be almost entirely genetic in nature. Model fits indicate that both pleiotropy and assortative mating contribute significantly and about equally to this genetic correlation.

## Author Summary

Traits that are attractive to the opposite sex are often positively correlated when scaled such that scores increase with attractiveness, and this correlation typically has a genetic component. Such traits can be genetically correlated due to genes that affect both traits and/or because assortative mating (people choosing mates who are similar to themselves) causes statistical correlations to develop between selected alleles across the traits. In this study, we used a large (total N = 7,905), genetically informative dataset to understand why two potentially sexually selected traits in humans—height and IQ—are correlated. We found that both shared genes and assortative mating were about equally important in causing the relationship between these two traits. To our knowledge, this is the first study that has been able to disambiguate the two principal reasons—shared genes versus assortative mating—for why traits can be genetically correlated.

## Introduction

Traits related to attractiveness are often positively correlated when scaled such that higher scores are more attractive. For example, personality traits of high novelty seeking, high reward dependence, and low harm avoidance are socially and sexually desirable [1], [2], and these traits are positively correlated when scaled in the same direction as attractiveness [3], [4]. Similarly, there are reports of positive correlations between the intelligence quotient (IQ) and health outcomes [5], physical and mental health [6], height and health [7], facial and bodily attractiveness [8], facial attractiveness and health [9], [10], facial attractiveness and IQ [11], and physical attractiveness and athleticism [12]. It is perhaps obvious but nevertheless important to note that such positive correlations are not a necessary consequence of each trait being related to attractiveness; in principal, it is perfectly plausible that the various components of attractiveness could be uncorrelated, or even often negatively correlated (implying trade-offs). That the empirical evidence suggests otherwise requires explanation.

There are two general types of explanations for consistent positive correlations between traits related to attractiveness. The first is that poor environments negatively affect these traits in the same direction. For example, poor nutrition in childhood is associated with worse health outcomes [13], lower IQ [14] and reduced height [15]. A non-mutually exclusive alternative for positive inter-correlations between traits related to attractiveness is that genetic effects are shared between such traits. This may occur either because the sets of genes affecting these traits partially overlap (*pleiotropy*) and/or because positive assortative mating for overall attractiveness causes cross-trait assortative mating, leading to genetic covariation due to *gametic phase disequilibrium*. The first of these factors, pleiotropy, is expected under “good genes” theories of sexual selection, whereby attractive features are honest signals of underlying genetic quality [16]. For example, sexually selected traits may be attended to precisely because they are sensitive to fitness-reducing mutations, and thereby reveal one's “mutational load.” On the other hand, to the degree that overall attractiveness is a composite of multiple traits, positive assortment between mates on attractiveness necessarily implies positive cross-trait correlations between traits that make up attractiveness. For example, if height and IQ were the only two traits differentiating people on attractiveness, then assortative mating on attractiveness would imply that smart people would mate not only with other smart people, but also with tall people at above chance levels. Assuming that such traits are heritable, cross-trait positive correlations cause a statistical relationship to develop between the ‘increasing’ alleles across the traits—gametic phase disequilibrium—thereby inducing a positive genetic covariation between the traits [17] (negative cross-trait assortative mating would induce a negative genetic correlation between traits). Such an increase in genetic covariation between traits under positive assortment mirrors the increase in genetic variation within traits that also occurs due to gametic phase disequilibrium.

In the present study, we investigate the nature of the correlation between two traits that appear to be under some degree of sexual selection in humans: IQ and height. Both sexes report that intelligence is among the most important qualities they look for in a mate [18], and consistent with the idea of assortment on a general “attractiveness” factor, there is a modest but consistent correlation between the IQ of mates [19]. Similarly, females prefer males who are ∼5 cm taller than average male height, whereas males prefer females ∼2 cm taller than average female height [20]. These average preferences are affected by one's own height, especially in males, who report desiring mates who are shorter than they are [20], [21], [22]. These preferences for height should also lead to positive assortment, as observed [23]. To the degree that smart females and tall males—and tall females and smart males—mate at levels above chance, it is possible that the correlation between height and IQ is due in part or in whole to cross-trait assortative mating. Alternatively, as predicted by “good genes” theories of sexual selection, it is also possible that some of the height-IQ correlation is due to genes that affect both. Finally, it is possible that the correlation between height and IQ is due to environmental influences that affect both. In the present study, we introduce a modeling approach that can distinguish between alternative explanations for why height and IQ—and traits related to attractiveness in general—are correlated.

### Previous research on the etiology of the height–IQ correlation

Taller people tend to be smarter. Although the relationship is modest, height and IQ are consistently correlated at ∼.10–.20 [24], [25], [26]. Three studies have examined the etiology of this correlation using a bivariate ACE “classical” twin design which uses the covariation of monozygotic (MZ) and dizygotic (DZ) twins to partition the variation in and covariation between height and IQ due to additive genetic effects (A), environmental factors shared on average between twins or siblings (C), and environmental factors that tend to affect individuals uniquely (E). Sundet et al. [24] found that the correlation between height and IQ in a sample of conscripted, Norwegian twin males was primarily due to shared environmental factors that explained 56% of the association, although overlapping genetic effects influencing both height and IQ were also significant and explained 35% of the association. Similarly, Beauchamp et al. [25] found that the height-IQ correlation was due to both shared environmental factors (explaining 59% of the association) and overlapping genetic factors (explaining 31% of the association) in a sample of Swedish twins. By contrast, Silventoinen et al. [26] found that overlapping genetic factors accounted for all of the covariation between height and IQ in four cohorts of Dutch twins. Differences between the samples may explain the inconsistency in conclusions: the twins investigated in by Sundet et al. [24] and Beauchamp et al. [25] were born after 1915 and 1886, respectively, whereas those investigated by Silventoinen et al. [26] were born after 1935 and mostly after 1980. It is likely that there were greater nutritional differences between families in the early 20^th^ century compared to the late 20^th^ century, which is consistent with substantially higher univariate estimates of shared environmental effects (∼20%) for both height and IQ in the former two studies compared to the latter study (0%). Thus, the architecture of the IQ-height correlation may itself vary between populations and time points.

A central limitation to all three previous studies investigating the height-IQ relationship is that assortative mating was not measured and accounted for. This is important for two reasons. First, if assortative mating occurs but is not modeled, shared environmental effects will be over-estimated and additive genetic effects under-estimated, as described by Eaves [27], [28] and shown graphically in Keller et al. [29]. As discussed in detail by Beauchamp et al. [25], this effect is not limited to biases in univariate effects: changing assumptions of cross-trait assortative mating has an equally dramatic influence on estimates of shared environmental and genetic effects on the correlation. If cross-trait assortative mating occurs but is not modeled, then what is actually a genetic correlation between the traits will *appear* as being due to shared environmental effects (despite the fact that assortative mating actually increases the *true* additive genetic variance/covariance). Thus, previous estimates showing the importance of shared environmental effects on the height-IQ correlation [24], [25] may have been biased upwards. The second reason that measuring and modeling assortative mating is important is that it allows researchers to estimate the degree to which any genetic correlation between traits is due to pleiotropy vs. gametic phase disequilibrium. Using a bivariate nuclear twin family design [27], researchers can determine whether the remaining additive genetic covariance is significant after accounting for the expected increase in additive genetic covariance between traits due to assortative mating.

### Present study

In the present study, we estimated the genetic and environmental influences on height and IQ using a bivariate nuclear twin family design, which models the covariation between MZ and DZ twins, their parents, and their siblings. The model we use here is described in Keller et al. [30], but owes its origins to models developed by Eaves and Heath [31], [32], [33] and Cloninger, Rice, and Reich [34], [35] in the 1970s and 1980s. As noted above, this model gives much less biased estimates of genetic and shared environmental effects than the classical twin design, estimates and accounts for the effect of assortative mating, and allows tests of the etiology of genetic correlations. Furthermore, the addition of siblings and parents greatly increases the precision of the estimates because adding more individuals within a family exponentially increases the amount of information on which estimates are based. For example, adding two siblings of a twin pair to the model provides five additional covariance estimates whereas adding another twin pair (also two individuals) provides only one additional covariance estimate [36].

## Results

### Phenotypic correlations

We analyzed height and IQ data from 2,936 families (n = 7,905) from four separate samples (see Methods). Table 1 shows descriptive statistics for IQ and height by sample, and Table 2 shows the phenotypic correlations between IQ and height on the combined data between various relative pair types. Within- and cross-trait correlations between MZ twins were roughly double those for DZ twins, suggesting important influences of additive genetic effects and minor influences of shared environments or genetic dominance on both IQ and height. However, as our results demonstrate below, such an inference can be wrong if genetic dominance and shared environments simultaneously influence variation in traits and if assortative mating is not accounted for [37], [38]. Correlations between spouses indicate that individuals mate assortatively on both height and IQ, and a cross-trait spousal correlation indicates that smart women partner with tall men (r = .18) and that smart males partner with tall women (r = .11). This pattern of spousal correlations suggests that a genetic correlation between height and IQ could have arisen as a result of cross-trait assortative mating, and not solely by genetic pleiotropy.

**Table 1:** Ns, Means, and Standard Deviations for raw IQ and height by sample.

			MZM	MZF	DZM	DZF	Bro.	Sis.	Fa.	Mo.
**LTS**	N		204	224	182	194	115	120	537	537
	IQ^a^	Mean	102.1	103.1	101.9	101.5	101.7	99.8	107.2	104.8
		SD	11.5	10.8	11.6	12.0	13.4	11.7	12.8	12.2
	HT^c^	Mean	175.8	163.8	176.5	165.4	169.9	165.1	180.3	165.6
		SD	7.9	7.1	7.9	6.9	10.2	7.4	6.9	6.9
**CTS**	N		410	518	533	543	205	197	-	-
	IQ^b^	Mean	10.6	9.9	10.9	10.0	11.1	10.2	-	-
		SD	2.4	2.4	2.5	2.3	2.4	2.4	-	-
	Ht^c^	Mean	177.8	165.1	178.8	164.8	178.8	166.4	-	-
		SD	8.6	7.6	7.9	7.1	9.1	6.9	-	-
**FAM**	N		-	-	-	-	574	202	268	353
	IQ^b^	Mean	-	-	-	-	10.9	10.1	11.1	10.7
		SD	-	-	-	-	2.6	2.2	2.3	2.5
	Ht^c^	Mean	-	-	-	-	173.7	161	177.8	163.8
		SD	-	-	-	-	11.4	8.9	7.1	7.1
**QIMR**	N		366	402	563	605	113	138	-	-
	IQ^a^	Mean	112.9	109.8	110.3	112.1	116.8	112.5	-	-
		SD	12.9	12.5	12.3	12.5	13.0	12.6	-	-
	Ht^c^	Mean	175	163.6	175.3	162.8	176.3	165.6	-	-
		SD	6.9	6.1	6.9	6.1	7.1	5.8	-	-

a Full scale IQ as measured by the WAIS/WISC;

b Average of Verbal+Performance IQ standardized subscales;

c Height in centimeters.

**Table 2:** Correlations by relative types in combined sample.

	IQ	Height	IQ - Height
**MZM**	.82	.85	.09
**MZF**	.80	.89	.13
**DZM**	.45	.43	.03
**DZF**	.54	.49	.10
**DZOS**	.45	.41	.07 (Bro IQ – Sis Ht).01 (Sis IQ – Bro Ht)
**BRO**	.46	.35	.04
**SIS**	.48	.47	.06
**BRO-SIS**	.42	.37	.08 (Bro IQ – Sis Ht).11 (Sis IQ – Bro Ht)
**FATHER**	–	–	.11
**MOTHER**	–	–	.22
**SPOUSE**	.35	.20	.11 (Male IQ – Fem. Ht).18 (Fem. IQ – Male Ht)
**FA-SON**	.40	.35	.07 (Son IQ – F Ht).11 (Fa IQ – Son Ht)
**FA-DAU**	.42	.43	.07 (Fa IQ – Dau. Ht).15 (Dau. IQ – Fa Ht)
**MO-SON**	.45	.33	.05 (Son IQ – Mo Ht).15 (Mo IQ – Son Ht)
**MO-DAU**	.44	.35	.11 (Dau. IQ – Mo Ht).17 (Mo IQ –Dau. Ht)

### Bivariate nuclear twin family design model fitting

**Figure 1:**
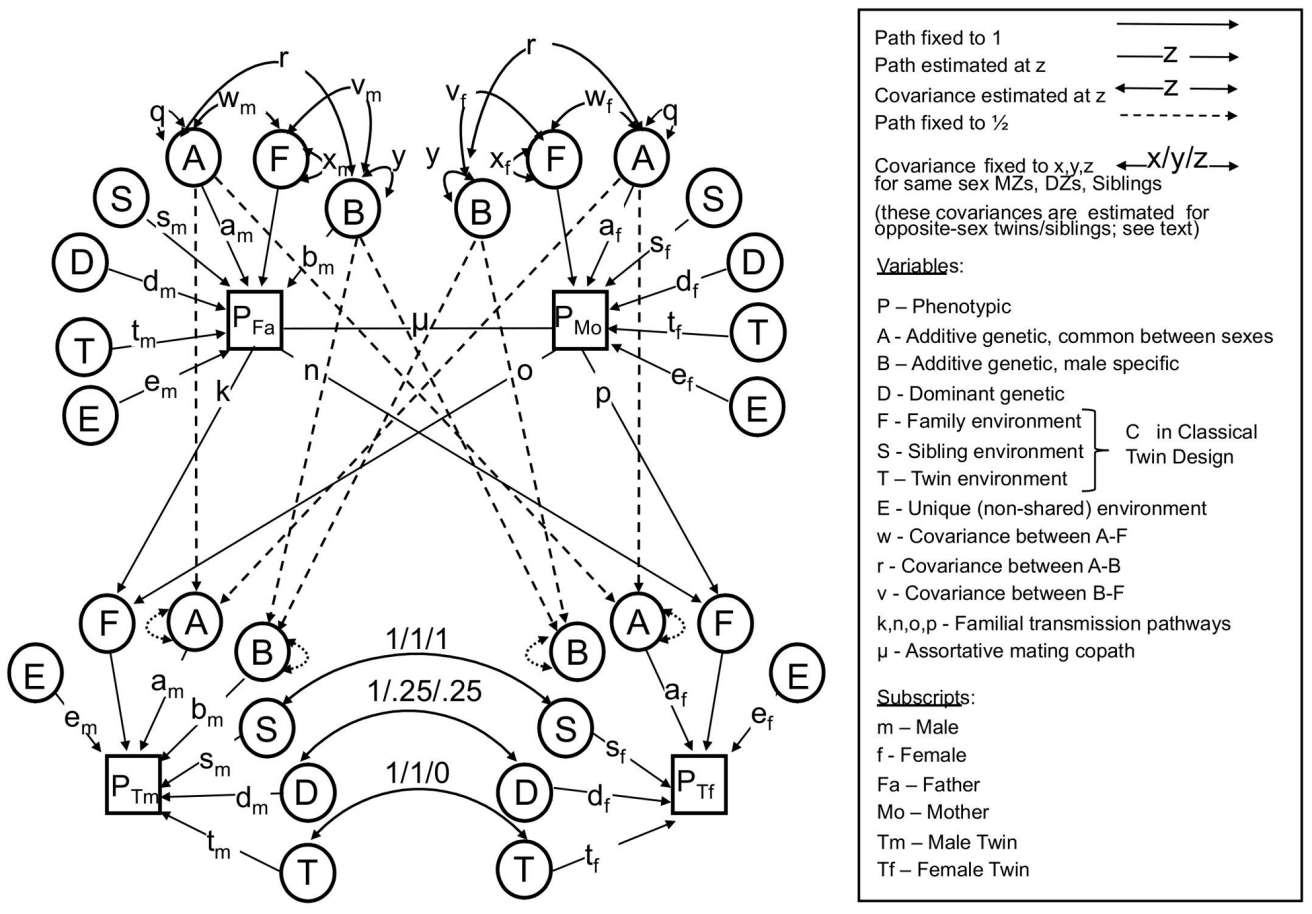
The full nuclear twin family design, with assortative mating modeled as primary phenotypic assortment. See text for descriptions of parameters. Note that either F or S must be dropped to make the model identifiable.

To formally model the relationship between height and IQ, we used the structural equation modeling framework first introduced by Sewall Wright [39], which has become the established approach in the behavioral genetics field. In particular, we used a bivariate nuclear twin family (NTF) design (see Methods and Figure 1) to model the sex-specific effects of the following influences: A - additive genetic effects shared in common between sexes; B – additive genetic effects specific to males (see below); D – dominant genetic effects arising from combinations of alleles at the same locus; S – sibling environmental effects arising from environmental factors shared between twins and siblings but not parents (e.g., school, peers, cohort, etc.); F - familial environmental effects arising from environmental factors passed from parents to children via “vertical transmission” (e.g., SES, education, etc.); T - twin environmental effects arising from environmental factors shared by twins, but not siblings or parents (e.g., classes at school, peers, prenatal environments); and E - unique environmental effects arising from factors that are unshared between relatives (e.g. unique experiences, measurement error, etc.).

**Figure 2:**
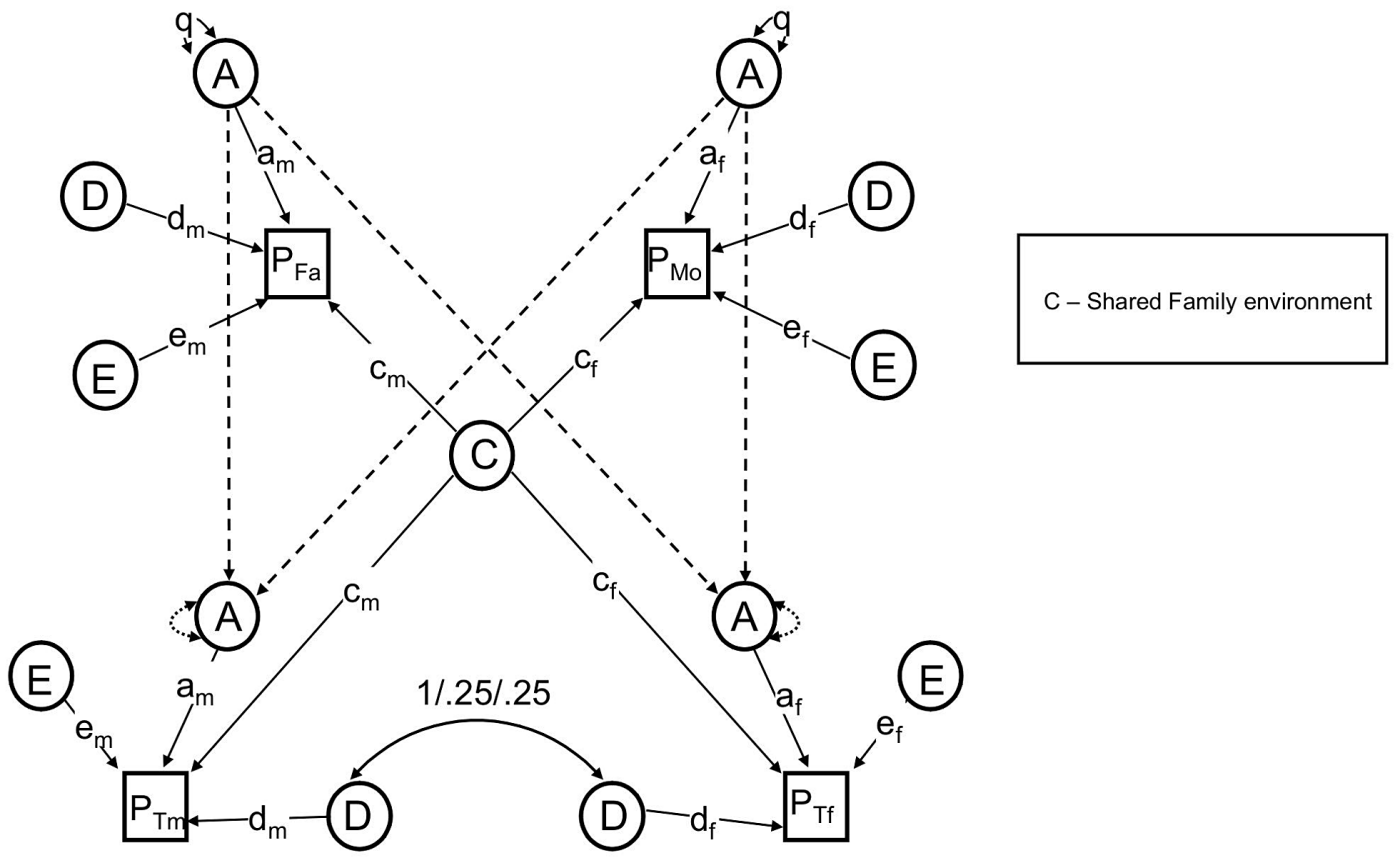
The reduced nuclear twin family design, with assortative mating modeled as social homogamy. All non-significant pathways and latent variables have been dropped.

The NTF design assumes that within-family similarity of non-genetic origin is due to either to parent/child vertical transmission (F) *or* to environments shared between siblings/twins but not shared by parents (S); a model estimating these two parameters simultaneously is not identified. We therefore fit two alternative primary phenotypic assortative mating models: an “ABDSTE” model and an “ABDFTE” model and compared their fits using the Akaike information criterion (AIC). The ABDSTE primary phenotypic assortment model (AIC = 8247.1) fit better than the ABDFTE primary phenotypic assortment model (AIC = 8250.0). Primary phenotypic assortment assumes that individuals actively choose similar mates based on their extant phenotype, but other causes of mate similarity are possible. The most commonly discussed alternative in humans is “social homogamy,” where similarity between mates arises from similar environmental backgrounds [33]. To gauge the degree of evidence for this hypothesis, we also tested an alternative “ABDCTE” NTF social homogamy model of assortment (see Figure 2), but it fit substantially worse than either of the primary assortative mating models (AIC = 8261.3), indicating that the process of primary phenotypic assortment (mates choosing similar mates) is most consistent with our data.

**Figure 3:**
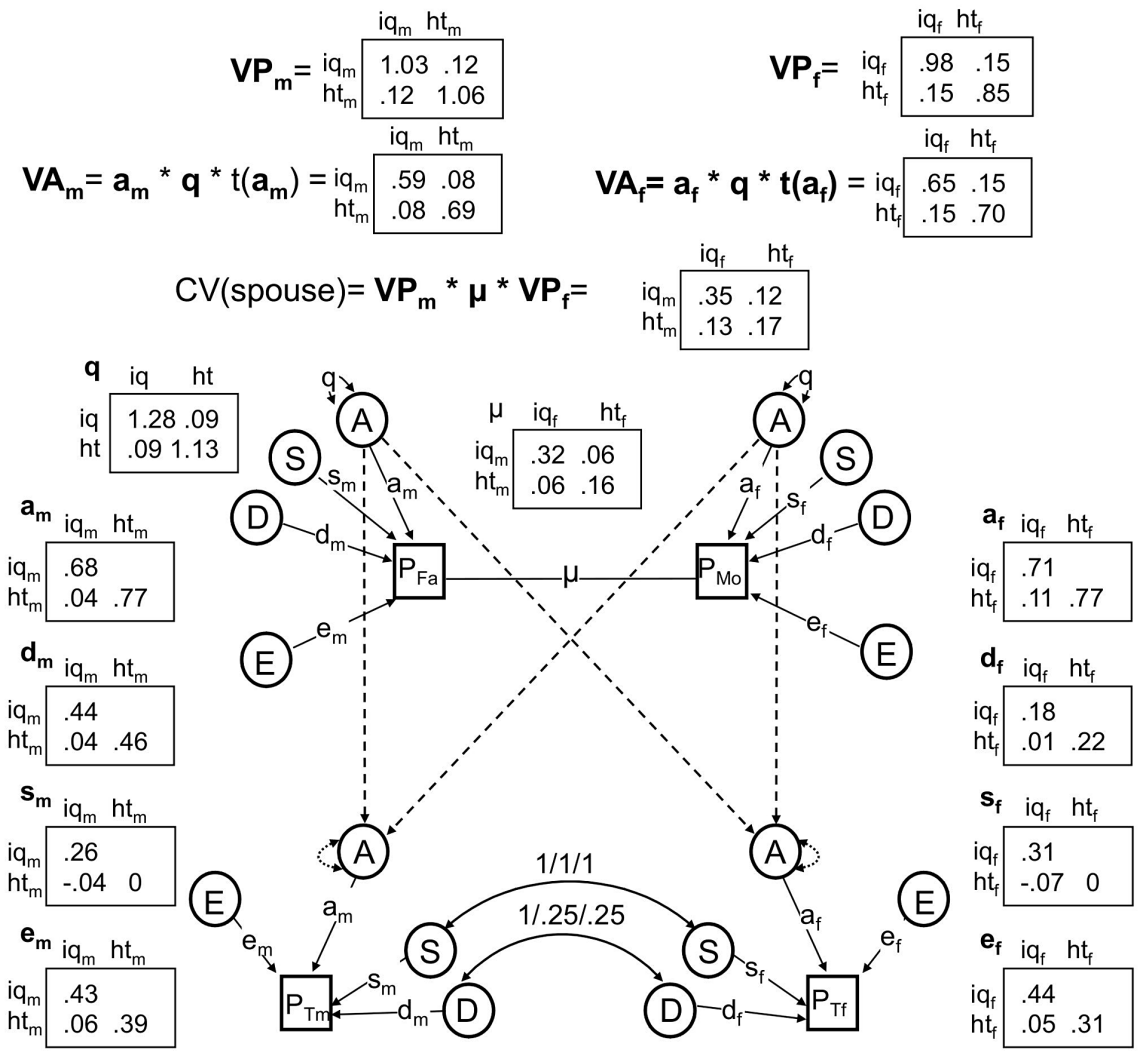
The best fitting nuclear twin family model, with assortative mating modeled as primary phenotypic assortment. All non-significant pathways have been dropped, and estimates of remaining pathways and latent variances are shown.

We sequentially dropped or fixed parameters in the ABDSTE model, beginning with those explaining the least amount of variance, until dropping additional parameters significantly reduced the fit of the model. Environmental effects unique to twins (T; χ^2^(6) = 7.8, *p* = .253) were non-significant, suggesting that environments shared by twins but not other siblings (e.g., teachers, peer groups) have at best minor effects on IQ or height. We also found no evidence for *qualitative* sex-limited effects (see Methods): (a) additive genetic effects unique to males (B) had almost no influence on model fit (χ^2^(3) = .202, *p* = .91), suggesting that the same genes affect IQ and height across the sexes; and (b) cross-sex correlations between both S (χ^2^(4) = 1.46, *p* = .83) and D (χ^2^(4) = 0, *p* = 1) could be fixed to 1, indicating that the same shared environmental and dominant genetic effects that influence male IQ and height also influence female IQ and height. No further parameters could be dropped or fixed. Environmental effects shared between siblings (S; χ^2^(6) = 16.99, *p* = .009), non-additive genetic effects (D; χ^2^(6) = 61.8, *p* = 1.9e-11), and additive genetic effects (A; χ^2^(6) = 1139.5, *p*<2e-16) were all highly significant factors influencing variation in IQ and/or height. A model dropping E could not be fit for technical reasons, although point estimates for E indicate its importance to model fit. This best-fitting, final model is shown in Figure 3 along with estimated variance components and coefficients for pathways.

Results from the final model indicated that the male narrow-sense heritability estimates of IQ (h_n_^2^ = .57) and height (h_n_^2^ = .65) were lower than corresponding narrow-sense heritabilities for females (h_n_^2^ = .67 and h_n_^2^ = .82 respectively). On the other hand, broad-sense heritabilities ([V_A_+V_D_]/V_P_) were similar between the sexes (h_b_^2^ = .75 for male IQ, h_b_^2^ = .85 for male height, h_b_^2^ = .80 for female IQ, and h_b_^2^ = .88 for female height). (It should be noted that these values are standardized by the respective phenotypic variances of males and females, and so are slightly different than the unstandardized values of V_A_ and V_A_+V_D_ shown in Figure 3). This indicates a greater influence of non-additive genetic effects in males than females for these traits. There were also modest influences of shared environmental effects on IQ for males (7% of the variation) and females (10% of the variation), but no such effects on height. Finally, most of the covariation between IQ and height was due to shared additive genetic influences: 68% for males and 100% for females. For males, unique environmental influences and dominant genetic influences appeared to play about equal roles in explaining the remaining covariation between IQ and height.

We also tested several questions related to assortative mating, which was modeled using a 2×2 full matrix of copaths between mates (μ) and a resulting change in additive genetic variance/covariance, modeled as a 2×2 full matrix of genetic variances/covariances, q (see Methods). Although there was stronger evidence that smart females pair with tall males (μ_12_ co-path = .08) than that smart males pair with tall females (μ_21_ co-path = .05), these two co-paths were not significantly different from one another (χ^2^(1) = .62, *p* = .43) and were constrained to be the same in the reduced model (estimated at μ_12_ = μ_21_ = .06). This co-path was significantly different from 0 (χ^2^(1) = 4.6, *p* = .03). Assortative mating inflates the level of additive genetic variation/covariation in the population, and the q matrix in our model quantified this increase. In particular, the additive genetic variation for IQ and height were 28% and 13% higher, respectively, than they would have been if couples mated at random (see the diagonals of the q matrix, Figure 3). Furthermore, by comparing the observed height-IQ covariance to the height-IQ covariance implied if q was an identity matrix, which it would be under random mating, we can conclude that the additive genetic covariance between IQ and height was much higher than it would have been under random mating: an estimated 167% higher in males (a predicted value of .03 under random mating vs. the observed value of .08) and 88% higher in females (a predicted value of .08 under random mating vs. the observed value of .15).

### The genetic correlation between height and IQ: Shared genes or assortative mating?

We estimate that the additive genetic correlation between height and IQ is .08 in males (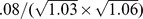) and .17 in females (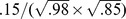), and these estimates were highly significant (χ^2^(3) = 47.4, *p* = 2.8e-10) . To understand whether pleiotropy (shared genes) was a significant cause of these correlations, we compared the final model to a model in which the off-diagonal paths in **a_m_** and **a_f_** were constrained to be 0; this model fit substantially worse (χ^2^(2) = 12.9, *p* = .002). Similarly, to understand whether gametic phase disequilibrium (assortative mating) was a significant cause of these correlations, we compared the final model to a model in which the off-diagonal elements of **q** were constrained to be 0; this model also fit substantially worse (χ^2^(1) = 14.4, *p* = .0001). These results give unequivocal support to the hypothesis that both shared genes and assortative mating are simultaneously responsible for the genetic correlation between height and IQ.

## Discussion

A positive correlation exists for many traits related to sexual attractiveness, as predicted by various evolutionary theories, but the true cause of this correlation is typically ambiguous. Here, we demonstrated how a genetically informative design that used twins, siblings, and parents can clarify the etiology of such correlations in humans. In addition, this design can provide estimates of the causes of variation in individual traits that are much more accurate and less biased than estimates from non-twin or twin-only designs. We used this model to demonstrate that the phenotypic correlation between two potentially sexually selected traits in humans, IQ and height, is largely genetic in nature, and that both shared genes and assortative mating contribute importantly to it. We believe that this approach can be used to systematically investigate the nature of correlations that exist between human traits related to attractiveness or to fitness in general.

An alternative approach that uses similarity at measured SNPs to estimate genetic relationships among classically ‘unrelated’ individuals has recently been used to estimate genetic correlations between traits [40], [41]. While the genetic association between height and IQ should be detectable using this method, it suffers from three limitations vis-à-vis the current approach. First, it would require much larger sample sizes than those used in the present study to detect genetic correlations of the magnitude observed here, because genetic relationships from distantly related individuals tend to have much less variance than those among twins and other family members. Second, heritability/genetic correlations estimated from similarity at measured SNPs only capture the effects of common (MAF>.01) causal variants, and so the covariance between IQ and height due to rarer mutations will not be detectable [42]. Finally, and most importantly, we are not aware of any way to directly estimate the relative contributions of assortative mating vs. pleiotropy on the genetic correlation when estimated from similarity at measured SNPs. The method described in this manuscript can disentangle the effects of pleiotropy from assortative mating because the degree of mate assortment is directly estimated and accounted for in the model.

The importance of genetic pleiotropy on the association between IQ and height is notable. On the surface, it might seem that height and IQ involve very different functional systems with different developmental origins. Genetic pleiotropy between IQ and height (indeed, between any two complex fitness traits) is consistent with the idea that variation in these traits partly reflects genome-wide mutational loads, and that these traits are components of attractiveness because of this—i.e., they are honest signals or cues of ‘good genes’ [43], [44], [45]. The additional and substantial increase in additive genetic covariance as a function of assortative mating is consistent with both traits being attractive to the opposite sex.

Because directional (including sexual) selection reduces additive genetic variation more quickly than non-additive genetic variation [46], [47], our results showing relatively higher levels of non-additive genetic variation in male height and IQ is consistent with the hypothesis that these traits have been under stronger selection in males than females. However, because the genes that affect these traits appear to be the same between males and females, selection for a trait in one sex would also lead to similar evolution of that trait in the other sex. Given that human mate choice is largely bi-directional, we might also predict that traits that males find particularly attractive in females should show higher levels of non-additive genetic variation in females than in males. Ironically, such depletion of additive genetic variation reduces their usefulness as indicators of ‘good genes,’ a situation known as the “lek paradox” [48]. A possible resolution to this is if sexually selected traits capture variation in overall condition [49], which is itself heritable due to, e.g., recurrent mutations that degrade condition [50].

Our univariate results are broadly consistent with what has been reported about the causes of phenotypic variation in IQ [51] and height [52] from previous studies: the causes of individual differences in these two traits are largely genetic in origin. However, our design does allow for less (downwardly) biased estimates of shared environmental influences, and we did detect significant albeit modest shared environmental effects on IQ (explaining ∼8% of variation) in both males and females. The effects of the shared environment on the genetic correlation between IQ and height were extremely small and negative, which may indicate a minor role of higher-order non-additive genetic effects on the genetic correlation rather than shared environmental effects per se actually causing dissimilarity between family members. It should be noted that any potential effects of population stratification on height and IQ would appear as positive shared environmental effects on the height-IQ correlation; our results therefore suggest such stratification has little if any effect on the estimated genetic correlation.

A limitation of the current study is that the results were based on a sample of different ages, from adolescence to late adulthood, and there is evidence that the genetic architecture of at least IQ changes over time, such that additive genetic influences become more pronounced whereas shared environmental influences decrease as individuals age [53]. It is therefore possible that effects of shared environments on IQ reported here are underestimated for adolescents and overestimated for older adults. Furthermore, as with almost all twin studies, the conclusions of our study rest on the assumption that environmental influences affecting IQ and height do not cause greater similarity in MZ twins than DZ twins. However, the possibility that this assumption is violated for these traits is increasingly unlikely in light of recent findings, also showing very high levels of additive genetic variation in height [42] and IQ [54], that are based on genomic similarity among unrelated individuals who are unlikely to share environmental factors. A final caveat to our results is worth consideration: it is likely that shared environmental influences play a larger role in height and IQ variation in cultures in which the relevant environmental factors (e.g., nutrition) vary to a greater extent between families. In such cultures, the proportionate effect, but not the absolute effect, of genes should be smaller than in the modern industrialized culture from which our samples were drawn.

In summary, this report has introduced an approach that can tease apart the three principal competing explanations (shared environments, shared genes, and assortative mating) for the etiology of correlations between sexually selected traits. We use this to conclude that both shared genes and the effects of assortative mating together account for most of the covariation between IQ and height. While other explanations cannot be excluded, our findings are consistent with the hypothesis that height and IQ are attractive because they tap into the same underlying factor of genetic quality—e.g., mutational loads—and that the resulting genetic correlation is accentuated by assortative mating on overall attractiveness. If so, we expect that many other traits related to attractiveness will also be genetically correlated due both to shared genes and to assortative mating. We hope that the current approach can serve as a template for testing this hypothesis across multiple traits related to human attractiveness.

## Methods

### Ethics statement

The study and protocols were approved by Institutional Review Boards at the University of Colorado and the Queensland Institute for Medical Research, and informed consent was obtained from all participants.

### Samples

Data from 2,936 families (n = 7,905) comes from four separate samples of individuals: the Colorado Longitudinal Twin Sample (LTS; 552 families; [55]), the Colorado Community Twin Sample (CTS; 1005 families; [55]), control subjects from the Colorado Adolescent Substance Abuse Family Study Sample (FAM; 401 families; [56]), and the adolescent twin sample from Queensland Australia (QIMR; 978 families; [57]). Together, these samples provide information from MZ and DZ twins (including same sex and opposite sex twins), parents of twins, and non-twin siblings (see Table 1). When samples were combined, ages ranged from 12 to 28 for twins, 10 to 35 for non-twin siblings and 29 to 78 for parents.

### Measures

For the three Colorado samples (LTS, CTS, and FAM), IQ was measured using the Wechsler Adult Intelligence Scale (WAIS-R or WAIS-III; administered to those over the age of 16; [58]) or the Wechsler Intelligence Scale for Children (WISC-R or WISC-III, administered to those 16 and under; [59]). In the CTS and FAM samples, and for the siblings in the LTS sample, scores on the verbal and performance subscales of the WAIS (or WISC) were averaged together to generate a measure of IQ. These two subscales together have been shown to correlate very highly with full-scale IQ [60]. In the LTS sample, full scale IQ was obtained from the WAIS-III or WISC-III. For the QIMR adolescent twin sample, IQ was obtained from a shortened version of the Multidimensional Aptitude Battery [61], which included three verbal subtests (Information, Arithmetic, Vocabulary) and two performance subtests (Spatial and Object Assembly). For all samples, we adjusted IQ for sex, age, and age squared. We also controlled for test version in the LTS and FAM samples (WAIS-III, WISC-III, WAIS-R, or WISC-R) because versions differed between individuals within these samples.

For all samples, height was self-reported. As with IQ, we adjusted scores of height to account for variance associated with sex, age, and age squared. For both height and IQ, we removed (set to missing) any scores that were more than 4 standard deviations above or below the mean because such outlying scores may have rare, non-familial causes (e.g., de novo mutations or environmental trauma). This affected a total of 10 scores: 8 negative outliers of height and 2 positive outliers of height (results were nearly identical when outliers were included). The final IQ and height scores in all samples were standardized residuals from our regression-based adjustments. Table 1 shows the sample sizes, means, and standard deviations for raw height and IQ scores by sample and relative type after outliers were removed. It should be noted that while overall phenotypic variance for both traits is, by definition, equal to one, variances within sex can be higher or lower than this.

### Bivariate nuclear twin family models

We used a bivariate nuclear twin family (NTF) design to model the variances of and covariances between MZ twins, DZ twins, their parents, and their non-twin siblings (Figure 1). For clarity, Figure 1 omits siblings (which are estimated exactly as DZ twins except that they do not share “twin environments”) and shows an example where twin 1 is a male and twin 2 a female. Each observed (squares) or latent (circles) variable in Figure 1 should be considered a 2-by-2 matrix of observed or latent scores of the effect in question, each covariance (double-headed arrows) a 2-by-2 full matrix of variance/covariance terms, and each pathway (single-headed arrows) a lower triangular matrix, specifying the pathways of a 2-by-2 Cholesky decomposition. The variance of each effect, derived by pre- and post-multiplying the pathway matrices by the variance matrices (which are identity matrices for all parameters except A, B, and F), gives the variance of the effect in question of IQ and height along the diagonals, and the covariance of the effect between IQ and height on the off-diagonals. Primary phenotypic assortative mating, denoted μ in Figure 1, is modeled as a 2-by-2 matrix of copaths [62], which has special rules associated with it, as described in Keller et al. [30].

The NTF design assumes that within-family similarity of non-genetic origin is due to either to parent/child vertical transmission (F) *or* to environments shared between siblings/twins but not shared by parents (S); a model estimating these two parameters simultaneously is not identified. We therefore fit two alternative primary phenotypic assortative mating models: an “ABDSTE” model and an “ABDFTE” model. Environmental factors causing twins to be more similar than non-twin siblings (T) could be estimated in all models. This model also assumes no effects of epistasis or gene-environment interactions. Nevertheless, the variance-covariance of D can be interpreted more broadly as reflecting any source of genetic non-additivity, including epistasis and gene-by-age interactions, as these influences tend to be captured by D in the NTF design [29]. We modeled *quantitative* sex-limited effects (for example, the same genes having different degrees of additive effects between sexes) using sex-specific pathways for A, D, S, T, and E. We modeled *qualitative* sex-limited effects of D, S, and T (for example, environmental factors causing twins/sibling similarity in females being different than those environmental factors doing so in males) by directly estimating a correlation between opposite-sex siblings/twins for these variables. Given our modeling approach for additive genetic effects, we had to model additive genetic *qualitative* sex limited effects (for example, height in males being affected by a different suite of genes than height in females) using male-specific additive genetic effects (B), as noted above. Modeling male-specific additive genetic effects was an arbitrary decision; modeling this as female-specific effects would not change the fit of the model or any conclusions. Finally, we estimated parent-offspring specific pathways (father-son, father-daughter, mother-son, and mother-daughter environmental transmission) for F. For a full explanation of this model, see Keller et al. [30].

Primary phenotypic assortment assumes that individuals actively choose similar mates based on their extant phenotype, but other causes of mate similarity are possible. The most commonly discussed alternative in humans is “social homogamy,” where similarity between mates arises from similar environmental backgrounds [33]. To gauge the degree of evidence for this hypothesis, we also tested an alternative “ABDCTE” NTF social homogamy model of assortment. In this model, shared environmental factors (**C**) contributed to covariance between all members within a family, including spouses and parents-offspring; S, F, and μ (primary phenotypic assortment) were therefore not estimated. Figure 2 shows this model with parameters found to be non-significant (see Results, below) omitted for clarity.

### Procedures

We estimated parameters using structural equation modeling on the four combined datasets using the raw data analysis option in OpenMx version 1.0.7. This script along with familial correlations that can be used to reproduce these results can be found at: www.matthewckeller.com. We first tested whether variances, covariances, and means of height and IQ could be equated between different types of relative; when they could not, we allowed them to differ in the model. We then ran the social homogamy model as well as two primary assortment models (ABDFTE and ABDSTE) and used the AIC to choose between these three non-nested models. We chose the best-fitting (lowest AIC) of these three models, and then began dropping non-significant parameters in that model one parameter at a time. Sequentially dropping parameters in a bivariate NTF design can be extremely burdensome due to the large number of parameters that can be tested and because of the dependency of significance on the order of the tests. Here, we adopted a common-sense approach to this, which dropped entire 2-by-2 matrices in an all-or-none manner if the fit of the model changed little after it was dropped (p>.10 on a χ^2^ test comparing -2 log likelihoods of the reduced model against the previous model). Given our liberal threshold (*p*<.10) for retaining parameters, a parameter (e.g., the **T** matrix, which has three free estimates, t_11_, t_12_, and t_22_) would only be dropped if there was little evidence for it in both height and IQ; strong evidence for either would result in retaining the parameter. We continued this process until no further parameters could be dropped. We tested whether *qualitative* sex-limited effects of S, D, and T existed by dropping the cross-sex correlations associated with these latent variables, and tested qualitative sex-limited additive genetic effects by dropping B (we did not attempt to constrain *quantitative* sex-limited effects). Finally, we investigated effects of assortative mating on model fits, testing whether genetic pleiotropy or assortative mating (or both) accounted for any observed genetic associations between height and IQ.
