# Correction: The Genetic Correlation between Height and IQ: Shared Genes or Assortative Mating?

**DOI:** 10.1371/journal.pgen.1004329

**Published:** 2014-03-27

**Authors:** The PLOS Genetics Staff

The first sentence under the heading “The genetic correlation between height and IQ: Shared genes or assortative mating?” in the Results section is incorrect and should read “We estimate that the additive genetic correlation between height and IQ is .13 in males 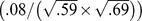 and .22 in females 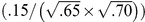, and these estimates were highly significant (*χ* ^2^(3)  =  47.4, *p* = 2.8e-10).”
